# Intergenerational Changes in the Waist Circumference and Selected Associated Indicators among Children and Adolescents from Kraków (Poland), between 1983 and 2020

**DOI:** 10.3390/ijerph20075344

**Published:** 2023-03-31

**Authors:** Łukasz Kryst, Magdalena Żegleń, Julia Badzińska, Agnieszka Woronkowicz, Małgorzata Kowal

**Affiliations:** 1 Department of Anthropology, Faculty of Physical Education and Sport, University of Physical Education in Kraków, 31-571 Kraków, Poland; 2 Pain Research Group, Institute of Psychology, Faculty of Philosophy, Jagiellonian University, 30-060 Kraków, Poland; 3 Doctoral School in the Social Sciences, Jagiellonian University, 30-060 Kraków, Poland

**Keywords:** secular trend, WHR, WHtR, waist circumference, adolescents

## Abstract

The aim of the study was to examine the direction of the secular changes in the waist and hips circumferences, as well as selected associated body proportions, among children and adolescents from Kraków, Poland. The study group included 8–18-year-olds examined in three cross-sectional studies (1983, 2010, and 2020). The analyzed characteristics included body height, circumferences of the waist and hips, which were used to calculate Waist-to-Hip Ratio (WHR), and Waist-to-Height Ratio (WHtR). There was a secular increase regarding the majority of the analyzed features, particularly for the younger children (i.e., prepubertal/early pubertal age). The trends were also especially evident when comparing the results of the 1983 series to the results of their peers examined in 2020. An opposite trend was noted in adolescent girls. The observed changes reflect the secular trend resulting from changes in body composition and fat distribution happening due to alterations in the lifestyle and socio-economic environment of the population over the years. It should also be stressed that the increase in the studied characteristics occurred mainly in younger children. This suggests that the observed changes may have resulted from a shift in the age of maturation and also from the personal and social motivators characteristic for late adolescence.

## 1. Introduction

In the last decade, a secular tendency towards a central deposition of fat tissue has been observed among Polish children [1]. Similar unfavorable trends regarding the prevalence of abdominal obesity as well as the intergenerational increase in waist circumference were also noted in a plethora of other populations [2,3,4,5,6].

Research has shown that excess fat tissue in the waist area can significantly elevate the risk of metabolic and cardiovascular diseases. Moreover, it can also be associated with an increased probability of abnormal levels of triglycerides, low-density lipoprotein, high-density lipoprotein, and insulin. Unfortunately, all of the mentioned risks apply not only to adults but also to children and adolescents. It should also be stressed that if central (abdominal) obesity occurs during childhood, there is a high probability of it persisting in later years of life, which makes it a significant public health issue [7,8,9,10,11].

A great way of assessing the risk of central obesity in the pediatric population is by using waist circumference, as well as its indicators, such as Waist-to-Height Ratio (WHtR) [12,13]. Another anthropometric indicator crucial for assessing the distribution of fat tissue and the risk of abdominal obesity is the Waist-to-Hip Ratio (WHR). It additionally includes the hips circumference, which, while not often used as a standalone parameter, is critical for calculating anthropological indicators, such as WHR [14].

Research taking into consideration the characteristics described above is fundamental due to the fact that the level of abdominal fat has been suggested to be a better predictor of cardiovascular risk compared to BMI (Body Mass Index) or general obesity, particularly in the pediatric population. Moreover, the BMI does not take into account the actual tissue composition of the body. Furthermore, the waist and hips circumferences have been noted to be significant predictors of intra-abdominal adiposity [10,14,15]. It should be stressed that in a recent study carried out among Polish preschoolers, it was also observed that WHR was preferable to Body Mass Index in assessing the risk associated with excess adiposity [16].

In the context of anthropometric studies, which often include fieldwork, screening studies, and are carried out in large populations, it is essential to mention that circumference measurements are relatively simple and inexpensive [14].

The aim of this study was to examine the direction of the secular changes in the waist and hips circumferences, as well as selected associated indicators, among children and adolescents from Kraków, Poland, in the years 1983, 2010, and 2020.

## 2. Material and Methods

The study group included 8–18-year-olds examined in three school-based, cross-sectional studies (1983, 2010, and 2020). The choice of research series was dictated by the availability of research material. In each series, the investigation was carried out in randomly selected schools (primary and high schools) in Kraków. The research on the growth and development of this population has a long history, starting before World War II. Unfortunately, not all series are fully compatible in terms of the assessed parameters and/or methodology. Thus, the cohorts have to be carefully chosen for particular research topics to facilitate the comparability of the results.

Consent was provided for the study from 2018 by the Bioethics Committee of the Regional Medical Association in Kraków (No 5/KBL/OIL/2019, 307/KBL/OIL/2019) as well as by the parents or legal guardians of the participants and the participants themselves.

All procedures contributing to this work complied with the ethical standards of the relevant national and institutional committees on human experimentation and the Helsinki Declaration of 1975, as revised in 2008.

The participants were classified into sex and age categories on the basis of their calendar age. The exact age was computed as a difference between the examination date and the birth date, expressed as a decimal fraction (e.g., the group of 9-year-olds included children aged 8.50 to 9.49).

The 1983 series consisted of 4993 children, and the 2010 and 2020 series included 2698 and 2167 participants (Table 1).

The analyzed anthropometric characteristics included circumferences of the waist and hips. They were measured with a nonstretchable anthropometric tape (accuracy: 5 mm). In addition, body height was measured using an anthropometer (accuracy: 1 mm; GPM, Switzerland), according to Martin’s method. The described anthropometric characteristics were used to calculate the following:

- Waist-to-Hips Ratio (WHR): [waist circumference/hips circumference];
- Waist-to-Height Ratio (WHtR): [waist circumference/body height].

In order to understand whether the interaction between sex, age category, and cohort differentiated the results for mean values of hip, circumference, waist circumference, WHR, and WHtR, a Generalized Linear Model (GLM) for multiple variables was used. Statistical analyses were performed using SPSS software v28.0.1.0 (IBM Corp, Armonk, NY, USA).

## 3. Results

### 3.1. Girls

**Figure 1:**
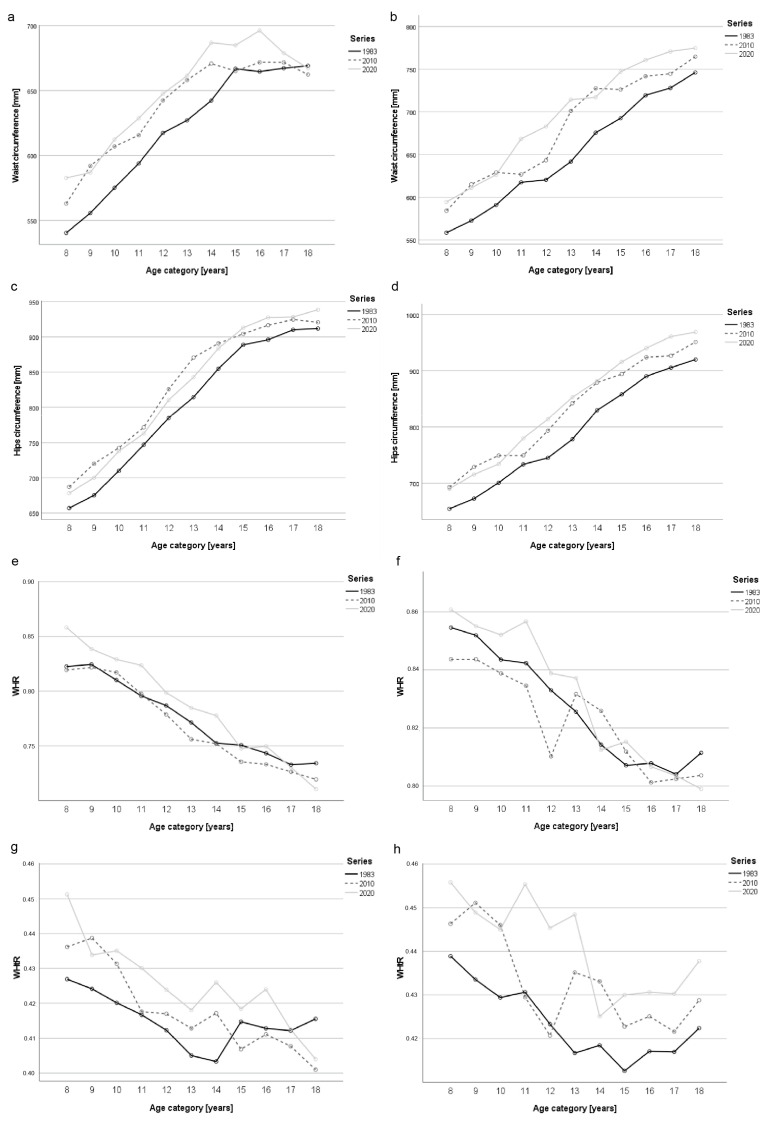
Changes in waist circumference ((**a**)—girls, (**b**)—boys), hips circumference ((**c**)—girls, (**d**)—boys), WHR ((**e**)—girls, (**f**)—boys), and WHtR ((**g**)—girls, (**h**)—boys) between the analyzed cohorts.

In the case of waist circumference, there was a statistically significant secular increase between 2020 and 1983 (the only exception was the group of 18-year-olds). A similar trend was also observed between 2020 and 2010 in most age categories; however, these differences did not reach statistical significance. The 2020 cohort was also generally characterized by a greater average hip circumference compared to their 1983 peers, and the differences were statistically significant. On the other hand, between 2020 and 2010, there was a secular decrease, but the observed differences were not statistically significant in most age groups (Table 2 and Table 3, Figure 1).

There was also an intergenerational increase (statistically significant in most age groups) of the mean value of WHR between 2020 and 1983. However, it should be mentioned that in some of the oldest girls, especially at 18 years of age, there was a decrease in the average WHR in the same period. In addition, except for the 18-year-olds, the 2020 cohort was generally characterized by a greater mean WHR compared to their peers measured in 2010, with most of the differences being statistically significant (Table 2 and Table 3, Figure 1).

Similarly to what was noted for WHR, the average values of WHtR were generally greater in the 2020 series compared to the 2010 and 1983 cohorts; the only exception was the group of oldest girls. The differences between 2020 and 1983 were statistically significant in most age groups.

The observed secular trends regarding the indicators based on trunk circumferences suggest that contemporary children and adolescents were characterized by a greater waist circumference not only in proportion to their waist girth but also in proportion to their body height compared to their counterparts examined in earlier years.

### 3.2. Boys

Similarly to what was noted among girls, there was a statistically significant secular increase regarding waist circumference between 2020 and 1983. Analogous intergenerational changes were noted in most age groups between 2020 and 2010, but the differences did not reach statistical significance. The children and adolescents examined in 2020 also generally had a greater mean circumference of the hips than that of their counterparts from the 1983 cohort, and the differences were statistically significant. A similar tendency was also present between 2020 and 2010 for most age groups, but the observed differences were statistically significant only in 11-, 12-, 15-, and 17-year-olds (Table 4 and Table 5, Figure 1).

Moreover, the average value of WHR was greater in the most contemporary cohort compared to the 2010 and 1983 series, except for the oldest age groups. However, the observed differences were not statistically significant in most age categories. Similarly to the results for WHR, the mean values of WHtR were, in most cases, greater in the 2020 series compared to peers examined in 2010 and 1983; the differences between 2020 and 1983 were statistically significant in most age categories (Table 4 and Table 5, Figure 1).

The observed intergenerational changes regarding the trunk-circumferences-based indicators imply that contemporary boys had greater waist circumference not only in proportion to their hip girth but also in proportion to their body height, compared to their peers examined in previous series.

**Table 2:** Descriptive statistics for girls—means and Standard Deviations (SDs) for the analyzed parameters.

Series	Age Category (y)	Waist Circumference (mm)		Hip Circumference (mm)		WHR		Body Height (mm)		WHtR	
		Mean	SD	Mean	SD	Mean	SD	Mean	SD	Mean	SD
1983	8	540.41	49.73	657.14	54.18	0.82	0.04	1266.00	55.01	0.43	0.04
	9	555.77	46.60	675.20	53.19	0.83	0.05	1309.00	57.83	0.42	0.03
	10	575.14	60.98	710.23	66.36	0.81	0.04	1369.00	64.67	0.42	0.04
	11	593.88	57.90	747.18	68.02	0.80	0.04	1425.00	71.15	0.42	0.04
	12	617.33	69.59	784.88	76.89	0.79	0.05	1496.00	72.25	0.41	0.04
	13	627.09	64.96	814.48	81.68	0.77	0.05	1547.00	67.94	0.41	0.04
	14	642.30	53.48	854.95	69.71	0.75	0.05	1593.00	57.85	0.40	0.03
	15	666.75	59.20	888.84	63.72	0.75	0.05	1608.00	54.59	0.42	0.04
	16	664.62	51.93	896.04	64.99	0.74	0.06	1611.00	56.06	0.41	0.03
	17	667.19	49.25	910.21	54.16	0.73	0.03	1619.00	57.27	0.41	0.03
	18	669.09	52.00	911.96	58.25	0.73	0.04	1611.00	59.08	0.42	0.03
2010	8	562.99	60.36	687.21	60.91	0.82	0.05	1291.00	60.56	0.44	0.04
	9	592.03	79.29	720.09	80.92	0.82	0.05	1348.00	62.46	0.44	0.05
	10	606.95	65.35	742.86	66.90	0.82	0.05	1409.00	62.24	0.43	0.04
	11	615.63	66.29	771.70	63.98	0.80	0.05	1477.00	65.69	0.42	0.04
	12	642.44	72.94	825.69	82.57	0.78	0.05	1541.00	71.26	0.42	0.04
	13	658.03	76.97	870.69	86.82	0.76	0.05	1593.00	58.70	0.41	0.05
	14	670.76	79.13	890.90	78.57	0.75	0.05	1609.00	61.46	0.42	0.05
	15	665.06	67.13	904.54	70.01	0.74	0.05	1638.00	56.73	0.41	0.04
	16	671.72	63.02	916.70	63.98	0.73	0.05	1636.00	61.95	0.41	0.04
	17	671.66	55.44	924.79	60.78	0.73	0.04	1649.00	51.11	0.41	0.04
	18	662.29	45.67	920.85	56.83	0.72	0.03	1656.00	57.99	0.40	0.03
2020	8	582.70	69.82	678.33	63.37	0.86	0.05	1292.00	58.47	0.45	0.05
	9	586.94	59.91	700.26	61.17	0.84	0.05	1352.00	64.97	0.43	0.04
	10	612.35	72.02	738.18	69.76	0.83	0.05	1408.00	71.50	0.44	0.05
	11	628.71	71.16	762.78	70.13	0.82	0.05	1462.00	74.56	0.43	0.04
	12	647.50	65.95	810.46	69.14	0.80	0.04	1528.00	72.90	0.42	0.04
	13	661.36	65.53	842.84	70.10	0.79	0.05	1584.00	56.34	0.42	0.04
	14	686.79	71.66	883.75	74.19	0.78	0.06	1616.00	73.51	0.43	0.05
	15	684.85	93.26	913.20	75.43	0.75	0.06	1641.00	55.59	0.42	0.06
	16	696.09	74.48	927.63	74.73	0.75	0.04	1644.00	52.43	0.42	0.05
	17	678.83	59.66	928.42	59.24	0.73	0.04	1644.00	49.78	0.41	0.04
	18	667.18	55.41	938.64	58.31	0.71	0.04	1655.00	57.19	0.40	0.03

WHR: Waist-to-Hip Ratio; WHtR: Waist-to-Height Ratio; SD: Standard Deviation.

**Table 3:** Differences between mean values of the analyzed characteristics between cohorts and age categories for girls.

Measurement	Series (I)	Series (J)	Difference in Means (I-J)										
			Age Category										
			8	9	10	11	12	13	14	15	16	17	18
Waist circumference (mm)	2010	1983	22.58 *	36.26 *	31.81 *	21.75 *	25.11 *	30.94 *	28.46 *	−1.67	7.10	4.47	−6.80
	2020	2010	19.71	−5.09	5.40	13.08	5.06	3.33	16.03	19.79	24.37 *	7.18	4.89
	2020	1983	42.29 *	31.17 *	37.21 *	34.83 *	30.17 *	34.27 *	44.50 *	18.10	31.47 *	11.64	−1.91
Hips circumference (mm)	2010	1983	30.07 *	44.89 *	32.62 *	24.52 *	40.80 *	56.21 *	35.95 *	15.67	20.66 *	14.58	8.89
	2020	2010	−8.87	−19.83	−4.67	−8.92	−15.23	−27.85 *	−7.15	8.66	10.93	3.63	17.79
	2020	1983	21.19 *	25.06 *	27.95 *	15.60	25.58 *	28.36 *	28.80 *	24.36 *	31.59 *	18.21	26.67 *
WHR	2010	1983	−0.003	−0.003	0.01	0.002	−0.01	−0.02 *	−0.001	−0.02 *	−0.01	−0.01	−0.02 *
	2020	2010	0.04 *	0.02 *	0.01	0.03 *	0.02 *	0.03 *	0.03 *	0.01	0.02 *	0.004	−0.01
	2020	1983	0.04 *	0.01 *	0.02 *	0.03 *	0.01	0.01	0.03 *	−0.003	0.01	−0.002	−0.02 *
WHtR	2010	1983	0.01	0.02 *	0.01	0.001	0.01	0.01	0.01 *	−0.01	−0.002	−0.004	−0.02 *
	2020	2010	0.02 *	−0.01	0.004	0.01	0.01	0.01	0.01	0.01	0.01	0.01	0.003
	2020	1983	0.02 *	0.01	0.02 *	0.01 *	0.01 *	0.01 *	0.02 *	0.004	0.01	0.001	−0.01

WHR: Waist-to-Hip Ratio; WHtR: Waist-to-Height Ratio; * the difference in means was significant (*p* < 0.05), based on estimated marginal means.

**Table 4:** Descriptive statistics for boys—means and standard deviations (SDs) for the analyzed parameters.

Series	Age Category (y)	Waist Circumference (mm)		Hip Circumference (mm)		WHR		Body Height (mm)		WHtR	
		Mean	SD	Mean	SD	Mean	SD	Mean	SD	Mean	SD
1983	8	558.52	52.61	654.43	58.13	0.86	0.05	1273.00	55.97	0.44	0.04
	9	572.64	51.33	672.37	53.63	0.85	0.04	1322.00	63.14	0.43	0.03
	10	591.10	63.21	700.73	62.29	0.84	0.04	1377.00	61.09	0.43	0.04
	11	617.47	57.72	733.55	60.65	0.84	0.04	1434.00	64.99	0.43	0.03
	12	620.43	59.68	745.10	63.14	0.83	0.05	1465.00	69.72	0.42	0.04
	13	641.78	59.22	778.25	67.05	0.83	0.05	1541.00	81.29	0.42	0.03
	14	675.64	74.35	829.52	74.68	0.81	0.04	1614.00	90.61	0.42	0.04
	15	692.60	64.79	857.93	63.89	0.81	0.04	1679.00	70.43	0.41	0.04
	16	719.53	63.23	890.19	59.69	0.81	0.04	1727.00	72.04	0.42	0.04
	17	728.06	51.01	905.48	47.39	0.80	0.04	1745.00	61.35	0.42	0.03
	18	746.22	50.67	920.00	50.38	0.81	0.04	1768.00	59.51	0.42	0.03
2010	8	584.18	64.70	692.68	67.34	0.84	0.05	1307.00	60.83	0.45	0.04
	9	615.19	82.93	728.84	83.59	0.84	0.05	1363.00	65.35	0.45	0.05
	10	629.14	81.49	749.36	77.56	0.84	0.05	1409.00	60.09	0.45	0.05
	11	626.83	107.72	749.44	102.58	0.84	0.05	1456.00	66.13	0.43	0.06
	12	643.40	74.22	793.15	67.87	0.81	0.05	1527.00	75.00	0.42	0.04
	13	701.21	96.38	842.16	89.06	0.83	0.05	1612.00	79.45	0.44	0.06
	14	727.65	105.56	878.91	93.20	0.83	0.06	1679.00	84.60	0.43	0.06
	15	726.27	83.87	893.99	79.69	0.81	0.05	1721.00	79.25	0.42	0.05
	16	741.77	96.92	923.97	83.96	0.80	0.05	1748.00	65.25	0.43	0.06
	17	744.77	83.83	926.71	75.62	0.80	0.05	1768.00	58.55	0.42	0.05
	18	764.76	81.95	951.21	76.42	0.80	0.05	1785.00	67.28	0.43	0.05
2020	8	594.47	69.52	690.15	61.44	0.86	0.05	1304.00	62.42	0.46	0.05
	9	611.03	63.40	715.56	67.61	0.86	0.06	1362.00	68.62	0.45	0.04
	10	626.56	79.15	734.10	71.69	0.85	0.05	1409.00	67.60	0.45	0.05
	11	668.48	85.81	779.65	76.50	0.86	0.05	1468.00	70.95	0.46	0.06
	12	682.96	87.56	813.73	85.15	0.84	0.05	1536.00	83.58	0.45	0.05
	13	714.30	90.12	852.83	86.26	0.84	0.05	1594.00	75.01	0.45	0.05
	14	717.28	86.74	881.80	78.77	0.81	0.05	1687.00	84.01	0.43	0.05
	15	747.04	97.27	915.81	89.93	0.82	0.05	1735.00	68.65	0.43	0.05
	16	760.71	107.99	940.24	80.60	0.81	0.06	1766.00	56.12	0.43	0.06
	17	770.88	67.97	960.88	68.53	0.80	0.05	1791.00	55.83	0.43	0.04
	18	774.84	66.99	968.87	60.07	0.80	0.04	1776.00	69.69	0.44	0.04

WHR: Waist-to-Hip Ratio; WHtR: Waist-to-Height Ratio; SD: Standard Deviation.

**Table 5:** Differences between mean values of the analyzed characteristics between cohorts and age categories for boys.

Measurement	Series (I)	Series (J)	Difference in Means (I-J)										
			Category										
			8	9	10	11	8	13	14	15	8	17	18
Waist circumference (mm)	2010	1983	25.66 *	42.55 *	38.04 *	9.36	22.96 *	59.44 *	52.01 *	33.66 *	22.24 *	16.70	18.54
	2020	2010	10.29	−4.16	−2.58	41.65 *	39.56 *	13.09	−10.37	20.78	18.95	26.12	10.08
	2020	1983	35.95 *	38.39 *	35.46 *	51.01 *	62.52 *	72.53 *	41.64 *	54.44 *	41.19 *	42.82 *	28.62
Hips circumference (mm)	2010	1983	38.25 *	56.46 *	48.63 *	15.90	48.05 *	63.91 *	49.40 *	36.06 *	33.78 *	21.22 *	31.21 *
	2020	2010	−2.53	−13.27	−15.26	30.21 *	20.58	10.67	2.88	21.81 *	16.27	34.18 *	17.66
	2020	1983	35.71 *	43.19 *	33.37 *	46.10 *	68.63 *	74.58 *	52.28 *	57.87 *	50.05 *	55.40 *	48.87 *
WHR	2010	1983	−0.01	−0.01	−0.01	−0.01	−0.02 *	0.01	0.01 *	0.01	−0.01	−0.002	−0.01
	2020	2010	0.02 *	0.01	0.01	0.02 *	0.03 *	0.01	−0.01	0.003	0.01	0.001	−0.01
	2020	1983	0.02	0.003	0.01	0.01 *	0.01	0.01	−0.002	0.01	−0.001	−0.001	−0.01
WHtR	2010	1983	0.01	0.02 *	0.02 *	−0.001	−0.003	0.02 *	0.02 *	0.01	0.01	0.01	0.01
	2020	2010	0.01	−0.002	−0.001	0.03 *	0.03 *	0.01	−0.01	0.01	0.01	0.01	0.01
	2020	1983	0.02 *	0.02 *	0.02 *	0.03 *	0.02 *	0.03 *	0.01	0.02 *	0.01	0.01	0.02

WHR: Waist-to-Hip Ratio; WHtR: Waist-to-Height Ratio; * the difference in means was significant at the 0.05 level, based on estimated marginal means.

### 3.3. Socioeconomic Characteristic of Examined Cohorts

The biggest change concerned the level of education. While in 1983 only 19.4% of mothers of surveyed children declared higher education, in 2010 it was already 56%, and in 2020 even 78%. In the case of the fathers of the surveyed children, it was 27%, 49%, and 66%, respectively (Table 6). Additionally, in 2020, the self-assessed financial situation of the family was better (7.05 on a 10-point scale), compared to the 2010 cohort (6.59 on a 10-point scale). Moreover, the number of children in the family decreased systematically from 2.43 in 1983, through 2.14 in 2010, to 2.04 in 2020.

## 4. Discussion

In the present study, there was a secular increase regarding the majority of the analyzed parameters, particularly for the younger children (i.e., prepubertal/early pubertal age). The trends were also especially evident when comparing the results of the 1983 series to the results of their peers examined in 2020.

Similar findings have been reported in other populations across a variety of age groups. For instance, research conducted in Great Britain showed a secular increase in waist circumference between 1990 and 2018. Interestingly, in the same study, it was suggested that the observed trends regarding waist circumference occurred, at least partially, independently of the intergenerational changes in BMI [17]. Additionally, in a recent study including Slovenian children, there was a secular increase in waist circumference and WHR and WHtR values, which also was in line with the current research results [18]. Moreover, in the Chinese population, there was an intergenerational increase in the value of WHtR, which concerned participants regardless of the level of urbanization of their place of residence (i.e., rural as well as an urban dwelling) [19].

The observed changes may be associated with secular trends regarding the distribution of fat tissue, as the tendency towards its central allocation was observed in Polish preschoolers [1]. A similar tendency has also been noted in 7–18-year-olds from Poland, as well as in Chinese adults [20,21].

Unfavorable changes in the distribution of fat tissue can stem from a variety of factors. For instance, it has been noted in the last few decades that there has been a significant drop in the level of physical activity, especially in youth [22]. Moreover, changes in the socioeconomic environment of the country can have a significant effect on the body composition and distribution of fat tissue in subsequent generations. In the 37 years dividing the studied cohorts, there was a decrease in the unemployment and at-risk-of-poverty rates, while the average salary, level of education, as well as GDP (Gross Domestic Product) increased [23].

Interestingly, in a study carried out in the Chinese population, the most significant secular increase in waist circumference and WHtR was found among 13–17-year-olds, compared to the younger age groups [24]. In contrast, in the present study, the most substantial increase of the analyzed parameters was generally noted for younger participants—at prepubertal and early pubertal age. The changes in waist-to-height value associated with age and puberty timing, which seem to exist in the present study, have previously been described in various populations, including in Greece, the USA, Pakistan, and Norway [25,26,27,28].

It should also be mentioned that the differences between the secular trends observed for varying age categories may be associated with personal (exploration of one‘s own identity) and social (peer pressure and sexual experimentation) motivators [28,29]. It may be the case that such factors have a more substantial role in the lives of more contemporary teens due to the popularity, variety, and widespread presence of social media. The pressure seems to affect contemporary adolescent girls in particular, as the change in direction of the observed secular trends was not present or was much weaker in boys. It should also be mentioned that analogous changes in the direction of intergenerational changes regarding body weight and BMI have already been described in this population [30]. Additionally, hip circumference depends on the development of the skeleton, gluteal musculature, and fat, so the effect of the secular trend is less pronounced. Body weight control in adolescent girls has previously been noted and attributed to social influences—mainly perceptions of friends’ dieting, types of conversations about body weight and dieting within the friendship group, as well as body comparisons [29,31].

In the context of the examined population, it should also be mentioned that Kraków’s population is quite heterogeneous. Thus, the results obtained in this group can be easily extrapolated to the general Polish population. On the other hand, it may also be a limitation, as the compared cohorts are not quite the same population. Therefore, the observed differences also express the mixing of genes with the immigrant population.

The secular increase in the average values of the majority of the analyzed characteristics may be associated with the changing socioeconomic environment in which each cohort was developing. For instance, the 1983 series included children and adolescents living in unfavorable conditions due to the political situation of the country, as well as the effects of the 1970s economic crisis. An improvement in the socioeconomic status of the Polish population can be observed only after the fall of communism, which happened in 1989. The analysis of these changes showed a clear trend, indicating a systematic improvement in the social and living conditions of society. The biggest change concerned the level of education, self-assessed financial situation, and the number of children in the family [32,33,34]. Later, significant economic progress and the following changes in the population’s lifestyle may have strongly influenced the intergenerational increase in trunk circumferences, as well as the values of the derived indicators [21,35].

## 5. Conclusions

In conclusion, there was a secular increase regarding most of the analyzed parameters between 1983 and 2020. The observed trends may be a result of changes in body composition and fat distribution happening due to alterations in the lifestyle and socioeconomic environment of the population occurring over the years. However, it should also be stressed that the intergenerational increase in the studied characteristics occurred mainly in children of prepubertal and early pubertal age. This suggests that the observed changes may be associated with a shift in the age of maturation and also with personal and social motivators characteristic for late adolescence.

## Figures and Tables

**Table 1:** Number of individuals and mean participants’ age in each of the examined cohorts.

Age Category (y)	1983		2010		2020	
	Average Age (y)	N	Average Age (y)	N	Average Age (y)	N
Girls						
8	7.54	194	7.98	102	8.06	126
9	8.51	201	8.97	111	8.99	117
10	9.49	214	9.98	98	9.97	162
11	10.58	209	10.99	96	10.97	167
12	11.51	232	11.94	80	11.90	108
13	12.54	220	13.10	102	12.96	88
14	13.52	196	13.95	187	14.03	92
15	14.52	246	15.00	162	15.00	100
16	15.48	247	16.00	172	16.01	80
17	16.56	217	16.92	163	16.89	60
18	17.52	252	17.98	120	18.48	55
Boys						
8	7.52	196	7.98	128	7.99	103
9	8.50	212	9.00	133	8.96	126
10	9.56	227	9.97	93	9.99	144
11	10.51	217	10.96	81	10.96	115
12	11.55	185	11.96	81	11.98	110
13	12.51	231	13.14	95	12.95	115
14	13.51	227	13.99	221	14.03	78
15	14.50	242	14.96	154	14.95	93
16	15.56	232	15.92	150	16.02	63
17	16.50	232	16.97	85	16.92	34
18	17.59	164	18.00	84	18.40	31

**Table 6:** Selected socioeconomic characteristics of the family.

Socioeconomic Characteristics of Family	Cohort		
	1983	2010	2020
Higher education—mothers (%)	19.4	56.0	78.0
Higher education—fathers (%)	27.0	49.0	66.0
Self-assessed financial situation *	-	6.59	7.05
Number of children in family (N)	2.43	2.14	2.04

* Scale 1 (very poor)—10 (very good).

## Data Availability

Research data are not shared. The data are not publicly available due to privacy or ethical restriction.
